# Factor structure and psychometric properties of the Body Perception Questionnaire–Short Form (BPQ-SF); The Persian version

**DOI:** 10.1371/journal.pone.0306348

**Published:** 2024-09-18

**Authors:** Simin Najari, Reza Rostami, Reza Kazemi, Hojjatollah Farahani

**Affiliations:** 1 Department of Psychology, University of Tehran, Tehran, Iran; 2 Faculty of Entrepreneurship, University of Tehran, Tehran, Iran; 3 Department of Psychology, Faculty of Humanities, Tarbiat Modares University, Tehran, Iran; Lahore School of Economics, PAKISTAN

## Abstract

**Background:**

Body perception is considered an important physiological marker in physical and mental disorders, therefore, its valid and reliable quantification is indeed necessary. Due to the lack of a Persian version of an instrument with validated psychometric properties for evaluating body perception, this study aimed to investigate the factorial structure, reliability, and validity of body perception questionnaire-Short Form (BPQ-SF) among Iranian adults.

**Methods:**

A total of 748 participants (mean age = 31.74; 57%female) were included in the analysis. Participants were recruited using the online survey method. Confirmatory factor analysis (CFA) was performed and concurrent validity was determined by computing Pearson’s correlation coefficient between BPQ_SF, Depression Anxiety Stress Scale (DASS), and somatization subscale of Symptom-Checklist-90 (SOM). Internal consistency (using Cronbach’s alpha and McDonald’s omega), and composite reliability were also evaluated.

**Results:**

The result of the CFA yielded three factors: Body Awareness, Subdiaphragmatic Reactivity, and Supradiaphragmatic Reactivity. Cronbach’s alpha values for all BPQ_SF items were 0.94. Moreover, Concurrent validity between BPQ, SOM, and DASS was determined to be between 0.44 to 0.94. and had good internal consistency (McDonald’s Omega range: .74-.93 Cronbach’s alpha range: .76-.94).

**Conclusions:**

The BPQ-SF demonstrated good psychometric properties among Iranians thus can be used to reliably assess body perception.

## 1. Introduction

Interoception, as the body-to-brain axis, monitors the body, receives the sensations concerning the internal state of the body, and transmits afferent information to the spinal cord and then to the brain. This information is processed in specific structures of the brain (e.g., anterior insula), and integration, monitoring, and awareness of the body occur along with the information coming from the systems engaged in attention, differentiation, and evaluation [1–4].

It is noteworthy that the subjective and objective aspects of interoception differ with the subjective experience stemming from internal body changes called body awareness or body perception [5]. Body perception consists of a broad spectrum of systems such as the cardiovascular, pulmonary, gastrointestinal, temperatures, genitourinary, pain, visceral, chemical sensory, immune, and autonomic nervous systems that contribute to maintaining body balance [2] via the sympathetic and/or the parasympathetic nervous system [6].

The polyvagal theory [7–10] provides a neurophysiological framework to investigate the autonomic nervous systems, distinguishing two branches of the tenth cranial nerve, each of which serves a specific adaptive behavioral strategy. The vagus nerve, which is the principal component of the autonomic nervous system, has two separate circuits in the parasympathetic nervous system: The ventral vagal complex (VVC)_to control the supra-diaphragmatic visceral organs_ and dorsal vagal complex (DVC)_to regulate the sub-diaphragmatic visceral organs_ which, together along with the sympathetic nervous system, influence one’s mental experiences and body awareness.

Interoception and body perception play a significant role in our emotional experiences, and their dysfunction is linked to many physical problems such as diabetes, obesity, and psychiatric disorders, such as depression [4, 11] generalized anxiety [3], autism spectrum disorder [12] and eating disorders [1, 13–15].

There are a limited number of self-report scales that can capture this important relationship between the brain and the body, and their psychometric properties have been confirmed [6]. However, according to Mehling et al. (2009), these instruments do not take the important areas of the body awareness structure into account and also come with psychometric limitations [16]. Although the Somatosensory Amplification Scale (SSAS) and the Modified Somatic Perception Questionnaire (MSPQ) have an acceptable psychometric foundation, there is, unfortunately, no consensus regarding their neurophysiological background [17].

The Body Perception Questionnaire (BPQ) was developed by Porges (1993b) to assess the mental experiences of the functions and reactions of the organs and structures that are innervated by the autonomic nervous system (according to the polyvagal theory). The BPQ is a self-report instrument whose initial version includes 122 items and five subscales, including body awareness, autonomic nervous system reactivity (supra-diaphragmatic reactivity (SUPR) and sub-diaphragmatic reactivity (SUBR)), cognitive-emotional-physical stress response, stress response styles, and health history. Since its development, the BPQ has been used in numerous studies. However, the wide use of this instrument has become limited partly due to the high number of questions. By extracting some aspects of the BPQ that received the most research interest, Cabrera et al. (2018) presented a shorter form (BPQ-SF) of this questionnaire. This 46-question version consists of body awareness and autonomic response subscales [6].

The body perception questionnaire-short form (BPQ-SF) is a self-report measure of two subscales with the first one being body awareness (26 items) measuring the sensitivity of bodily signals (e.g. “During most situations, I am aware of how fast I am breathing.”). The autonomic nervous system reactivity subscale which is composed of a Supradiaphragmatic Reactivity (SUPR; 15 items) which measures the responses of autonomically innervated organs above the diaphragm and reflects the unique effects of ventral vagal complex (e.g. “During stressful situations, I am aware that My heart often beats irregularly.”) and a subdiaphragmatic reactivity (SUBR; 6 items) which measure the responses of autonomically-innervated gastrointestinal organs below the diaphragm and reflect dorsal vagal complex (according to the polyvagal theory) (e.g. “During stressful situations I am aware that I have indigestion”) is the second subscale. It is worth noting that Question 41 is common to both SUPR and SUBR subscales.

The BPQ-SF has been translated into several languages so far. In a study by Wang et al. (2020), the BPQ-SF psychometric properties were assessed in a sample of 688 Chinese university students and the three-factor structure of this questionnaire was approved, and its convergent and divergent validity were reported to be desirable [18]. Moreover, the total internal consistency was found to be 0.94, and the test-retest reliability 0.78. Poli et al. (2021) investigated the BPQ-SF psychometric properties in an Italian population and found an acceptable factor structure and validity with a test-retest reliability of above 0.70, and a correlation coefficient for each of the subscales of body awareness and the autonomic nervous system reactivity (SUPR and SUBR) of 0.79, 0.78, and 0.77, respectively [17].

Given its important role in the emergence and occurrence of many psychiatric disorders, the study of body perception can be insightful, and considering the lack of the BPQ Persian version, the present study aimed at standardizing the concurrent validity and the internal consistency of the Persian version of the BPQ-SF.

## 2. Materials and method

### 2.1 Participants

A total of 748 people (Age range = 18 to 77; Mean = 31.74, SD = 51.32; 57% female) participated online in the present study in response to a public call (December 2021 to January 2022).

Questionnaires were created through online testing platforms (Google Forms) and were provided to the participants through a link. Volunteers were included in this research if they met the inclusion criteria such as a having a minimum age of 18 and absence of psychiatric and chronic medical disorders.

The online questionnaire was made available to the students of the University of Tehran on social networks and they were asked to share it with other volunteers. Therefore, the data collection can be considered to have been a non-random snowball sampling method. There was no financial incentives for participation and the quality of the data was checked by a psychologist.

At first, a brief explanation of the research goals, objectives, tools, and the needed response time was provided. By accepting and participating in this project, demographic information was first collected from the participants, including age, gender, education, marital status (Single or Married), occupation, as well as any diagnosis and use of certain medical and psychiatric drugs. Participants diagnosed with any psychiatric conditions or taking psychiatric medications were excluded from the study. Considering that answering every question was mandatory to be able to proceed to the next question, there was no missing data in this study.

To estimate the sample size, according to [19], the ratio of subjects to items (N: P) was considered to be about 15. Considering that the questionnaire has 46 items and considering a 10% dropout, the sample size was determined to be 759 people. In the end, 736 subjects were included in the final analysis after removing problematic answers.

### 2.2 Procedures

First, the translation and cultural adaptation of the BPQ-SF were performed. The BPQ-SF was translated by three psychologists, all fluent in Persian and English. The Persian version was assessed by expert psychologists and then back-translated into English by two professional translators. The two versions were compared regarding content and translation quality and no fundamental difference was observed between the original and the back-translated version. This study was approved by the University of Tehran’s Ethics Committee. All participants gave their written informed consent to participate in the research.

### 2.3 Measures

#### The Body Perception Questionnaire- Short Form (BPQ-SF)

BPQ-SF is a self-report (46 items) measure with three subscales: Body awareness (BA), Supradiaphragmatic reactivity (SUPR), and subdiaphragmatic reactivity (SUBR). Strong psychometric properties and a constant factor structure across different languages have been reported. The BPQ-SF is answered on a five-point Likert scale ranging from 1 = never to 5 = always.

#### The Symptom Checklist 90 (SCL-90) somatization subscale (SOM)

The SCL-90 [20], as a self-report instrument, includes 90 items and nine subscales to measure symptoms of mental distress using a five-point Likert scale ranging from not at all = 1 to severe = 5. Among its nine subscales, scores of the somatization subscale (SOM) were analyzed in this study. SOM has 12 questions to measure discomfort stemming from the perception of the body’s unhealthy function and has been previously validated in an Iranian sample with its internal consistency (Cronbach’s alpha) and test-retest reliability reported at 0.87 and 0.77, respectively [21].

#### The Depression Anxiety Stress Scales (DASS-21)

The DASS (Lovibond, 1995) is a 21-item self-report questionnaire that assesses the negative emotional depressive states (including dysphoria, hopelessness, devaluation of life, self-depreciation, lack of interest/involvement, anhedonia, inertia), anxiety (including autonomic arousal, skeletal muscle effects, situational anxiety, subjective experience of anxious affect), and stress (including difficulty relaxing, nervous arousal, being easily upset/agitated, irritable/over-reactive) with 7 questions each. Participants are asked to rate the severity of the symptoms over the past week on a four-point Likert scale from not at all = 0 to very much = 3 [22]. The internal consistency (Cronbach’s alpha) of the Persian version in the Iranian population was reported at 0.77 for the depression subscale; 0.79 for the anxiety subscale; and 0.78 for the stress subscale [23].

### 2.4 Data analysis

Data analysis was conducted using R version 4.3.1 (R Core Team 2017) and SPSS version 4.2 (SPSS Inc., Chicago, IL), and a significance level of 0.05 was considered significant for all the analyses. The data set was complete with no missing values, allowing for analyses to be performed on the entire data set.

#### Descriptive statistics

Were used to summarize the study variables and demographic characteristics of participants. Then, the psychometric properties of the BPQ-SF scale were tested. Ceiling and floor effects were checked using a cut-off point of 15%, as recommended by [24]. The Lavaan [25] package in RStudio version 4.2 was used for this purpose.

#### Factor analysis

Confirmatory factor analysis (CFA) was conducted to test the fit of the originally proposed factor structure of the BPQ-SF. The diagonally weighted least squares (DWLS) was used as the estimation method. This method is preferred when dealing with categorically observed variables and when multivariate normality is not met [26, 27] as it provides more accurate factor loadings and it can have superior control over Type I error rates [28–30]. Model fit was assessed using several fitness indices, including chi-square and its degrees of freedom (p >.05), the comparative fit index (CFI >0.90; [31]), Tucker–Lewis Index (TLI >0.90; [31]), and the root mean square error of approximation (RMSEA) with 90% confidence interval (RMSEA >0.08). After confirming the factor analysis, measurement invariance was carried out to assess whether the scale is invariant across gender groups. Three models were constructed: a configural model (to assess the similarity of factor loading patterns across the groups), a metric model (to assess the equality of factor loadings across groups), and a scalar model (to assess the equality of intercepts across groups).

#### Measurement Invariance Analysis (MIA)

To examine the measurement invariance (MI), model comparisons were made between configural and metric models on one side, and between scalar and metric models on the other side. MI was supported if model comparisons showed ΔCFI <0.01, ΔRMSEA <0.015, and ΔTLI <0.01 [32–34].

#### Internal consistency

To determine reliability, an internal consistency approach was applied thus Cronbach’s alpha and McDonald’s Omega were determined for the total questionnaire and also for each factor with a threshold of >0.7 was considered acceptable [35]. The diagonally weighted least squares (DWLS) was used as the estimation method. The correlation coefficient of the item score and the total correlation of the corrected items were also calculated.

#### Concurrent criterion validity

Pearson’s correlation coefficients of the BPQ, SOM, and DASS scores were determined to assess evidence of validity. After calculating Pearson’s correlation coefficients, the questionnaire items and total scores were assessed to evaluate the validity of the investigated criterion. For convergent validity, Average Extracted Variance (AVE) and Composite Reliability (CR) were considered.

Kline (2023) believed that AVE >0.5 indicates a high level of convergent validity of a scale. In addition to AVE, when CR exceeds 0.8, a scale has a good evidence of convergent validity [19].

## 3. Results

The demographic information is presented in Table 1. It is noteworthy that the questionnaire raw data is provided in supporting information section S1 Data as an excel file.

**Table 1:** Demographic information for all participants (N = 748).

Variable	Level	f	(%)
Sex	Female	426	(57.0)
	Male	322	(43.0)
Marital status	Single	514	(68.7)
	Married	234	(31.3)
Education	Diploma & under	61	(8.2)
	A. D	36	(4.8)
	B.A/S	303	(40.5)
	M.A/S	289	(38.6)
	PhD	59	(7.9)

*Note*. f = frequency. A.D = Associate Degree; B.A = Bachelor of Arts; B.S = Bachelor of Science; M.A = Master of Arts; M.S = Master of Science; PhD = Doctor of Philosophy.

The majority of participants (68%) were single and female (57%). Also, most of the participants had an academic education.

**Fig 1:**
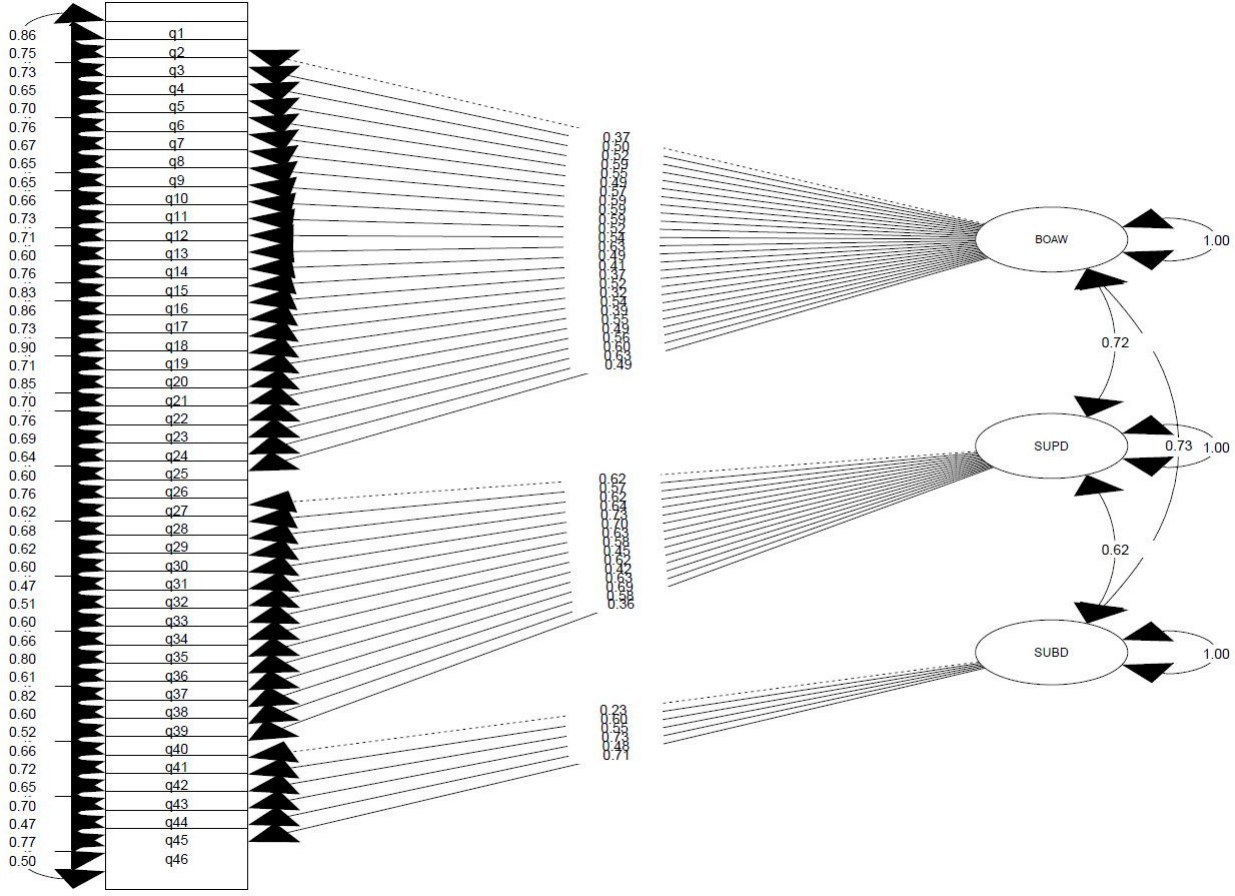
Coefficients path and the CFA BPQ-SF Persian model.

The results of CFA indicated that the originally proposed factor structure of BPQ-SF provided a good fit (RMSEA = 0.046; CFI = 0.973; TLI = 0.958). As shown in Table 2, item loadings were above the minimum threshold of 0.3 and significant. As a result, all of items showed acceptable factor loadings. Therefore, the main factor structure was confirmed and each item was placed in its own factor. With the difference that in the original form BPQ-SF, question number 41 was jointly loaded on both the second and third factors, but in our study, according to the fit indices, this item was placed in the third factor, because by placing this item in the third factor, the indicators had a more favorable state. Fig 1 shows how the items are placed in each of the factors. It is worth mentioning that the total score was calculated across all the items and the scores of each subscale from the sum of the scores of the items placed in the same subscale.

**Table 2:** CFA and CISs of Body Perception Questionnaire-Short Form (BPQ_SF).

Item	B	se	Beta	t	P	CICs	
						
**Body awareness**							
q1. Swallowing frequently	1.000		0.14	0.37		0.34	
q2. An urge to cough or clear my throat	1.37	1.44	0.50	9.52	<0.001	0.47	
q3. My mouth being dry	1.4	0.15	0.52	8.9	<0.001	0.48	
q4. Breathing is faster	1.57	0.17	0.6	9.42	<0.001	0.55	
q5. Watering or tearing of my eyes	1.54	0.17	0.55	8.84	<0.001	0.51	
q6. Noises associated with my digestion	1.38	1.84	0.5	7.51	<0.001	0.46	
q7. A swelling of my body or parts of my body	1.4	0.17	0.57	8.01	<0.001	0.53	
q8. An urge to defecate	1.77	0.2	0.59	8.84	<0.001	0.54	
q9. Muscle tension in my arms and legs	1.82	0.21	0.59	8.81	<0.001	0.54	
q10.A bloated feeling because of water retention	1.80	0.16	0.51	6.98	<0.001	0.55	
q11. Muscle tension in my face	1.13	0.16	0.51	6.98	<0.001	0.50	
q12. Goosebumps	1.44	0.17	0.54	8.46	<0.001	0.51	
q13.Stomach and gut pains	2.07	0.22	0.63	9.04	<0.001	0.59	
q14.Stomach distension or bloatedness	1.56	0.18	0.49	8.37	<0.001	0.47	
q15. Palms sweating	1.37	0.18	0.41	7.38	<0.001	0.38	
q16. Sweat on my forehead	1.15	0.16	0.37	7.06	<0.001	0.35	
q17. Tremor in my lips	1.06	0.15	0.52	7.01	<0.001	0.50	
q18. Sweat in my armpits	1.01	0.14	0.32	6.95	<0.001	0.31	
q19. The temperature of my face (especially my ears)	1.53	0.19	0.54	7.82	<0.001	0.50	
q20. Grinding my teeth	1.03	0.15	0.39	6.61	<0.001	0.36	
q21. General jitteriness	1.78	0.21	0.54	8.28	<0.001	0.51	
q22. The hair on the back of my neck standing up	0.98	015	0.5	6.28	<0.001	0.47	
q23. Difficulty in focusing	1.77	0.2	0.55	8.61	<0.001	0.52	
q24. An urge to swallow	1.66	0.20	0.60	8.19	<0.001	0.56	
q25. Heartbeat has increased	1.82	0.20	0.62	8.71	<0.001	0.58	
q26.Feeling constipated	1.54	0.19	0.49	8.05	<0.001	0.47	
**Supradiaphragmatic Reactivity**							
q27. I have difficulty coordinating breathing and eating	1.00		0.62			0.52	
q28. When I am eating, I have difficulty talking	1.01	0.07	0.57	13.78	<0.001	0.48	
q29. My heart often beats irregularly	1.30	0.10	0.61	12.06	<0.001	0.52	
q30. When I eat, food feels dry and sticks to my mouth and throat	1.09	0.08	0.63	13.47	<0.001	0.53	
q31. I feel shortness of breath	1.57	0.11	0.72	13.31	<0.001	0.60	
q32. I have difficulty coordinating breathing with talking	1.49	0.11	0.70	12.89	<0.001	0.58	
q33. When I eat, I have difficulty coordinating swallowing, chewing, and/or sucking with breathing	0.98	0.07	0.63	13.46	<0.001	0.53	
q34. I have a persistent cough that interferes with my talking and eating	0.83	0.08	0.58	9.87	<0.001	0.49	
q35. I gag from the saliva in my mouth	0.54	0.08	0.45	9.87	<0.001	0.40	
q36. I have chest pains	1.12	0.10	0.62	10.70	<0.001	0.52	
q37. I gag when I eat	0.51	0.07	0.42	6.91	<0.001	0.80	
q38. When I talk, I often feel I should cough or swallow the saliva in my mouth	1.17	0.10	0.63	11.31	<0.001	0.53	
q39. When I breathe, I feel like I cannot get enough oxygen	1.47	0.11	0.68	13.11	<0.001	0.57	
q40. I have difficulty controlling my eyes	0.93	0.08	0.57	10.93	<0.001	0.49	
**Subdiaphragmatic reactivity**							
q41. I feel like vomiting	0.63	0.11	0.35	5.41	<0.001	0.48	
q42. I have sour stomach	3.29	0.97	0.59	3.37	<0.001	0.46	
q43. I am constipated	3.33	1.00	0.54	3.33	<0.001	0.44	
q44. I have indigestion	3.62	1.06	0.72	3.39	<0.001	0.56	
q45. After eating i have digestive problems	2.21	0.67	0.48	3.27	<0.001	0.37	
q46. I have diarrhea	3.74	1.12	0.70	3.34	<0.001	0.55	

According to the results of the Merdia test as shown in Table 3, skewness was significant, so the DWLS method is used, which has more robustness. The model was found to fit well the first time, and there was no need to correct the multicollinearity between the items, and there were no visible outliers.

**Table 3:** Mardia’ test result.

Test	statistic	pValue
Mardia skewness	44905.01	<0.001
Mardia kurtosis	1222.88	<0.001

Absolute fit index such as RMSEA, SRMR, and CFI were used as comparative fit index (CFI) in model fitting. as the Chi-square value is an index sensitive to sample size (Kline 2023). Therefore, its normalized ratio (divided by the degree of freedom) was used, with a value less than 3 indicating a good fit of the model [19]. In this study, the Chi-square value with the degree of freedom of 985 was equal to 2027.42 and the normalized value was equal to 2.058 which is acceptable based on literature [36]. A RMSEA greater than 0.10 indicates poor fit [37]. The RMSEA value in our study was 0.038, indicating the model’s good fitness. The values of other fit indexes including the goodness of fit index (GFI), comparative fit index (CFI), normed fit index (NFI), non-normed fit index (NNFI), incremental fit index (IFI) and adjusted goodness of fit index (AGFI) were 0.96, 0.97, 0.94, 0.97, 0.97 and 0.95, respectively. These indices indicate a very good fit, if greater than 0.9, and a good fit, if greater than 0.8 [38]. Since they were both greater than 0.89, a good fit of the model was achieved (Table 4).

**Table 4:** Goodness of fit indices.

Df	X^2^	P	X^2^/df	RMSEA	SRMR	CFI	IFI	NFI	NNFI	GFI	AGFI
**985**	2027.42		2.058	0.038	0.062	0.97	0.97	0.94	0.97	0.96	0.95

Next, a Multigroup CFA using the DWLS estimator was performed to test if the factor structure of the BPQ-SF is invariant across gender groups. As shown in Table 5, the configural model of multigroup CFA demonstrated acceptable fits based on all three fit indices. Furthermore, constraining factor loadings being equal across genders revealed that ΔCFI, ΔRMSEA, and ΔTLI were within the suggested cut-offs, representing metric invariance. At last, constraining item intercepts to be equal across genders revealed that ΔCFI, ΔRMSEA, and ΔTLI were within suggested cut-offs, representing scalar invariance. Table 5 reports results from Measurement Invariance Analysis.

**Table 5:** Gender measurement invariance analysis of BPQ.

Robust Model Fit Indexes									Model differences		
model	X^2^	df	*p*value	CFI	TLI	RMSEA	SRMR	ΔX^2^	ΔCFI	ΔRMSEA	ΔTLI
M1	6180.823	1970	<0.001	0.697	0.682	0.076	0.67	---	----	-----	----
M2	6250.657	2014	<0.001	0.695	0.687	0.075	0.72	69.83	0.002	0.002	0.005
M3	6567.016	2057	<0.001	0.676	0.674	0.77	0.74	386.19	0.021	0.1	0.008

### 3.1 Descriptive statistics

Descriptive statistics for the BPQ-SF and DASS subscales and SOM are shown in Table 6.

**Table 6:** Descriptive statistics of BPQ-SF, DASS and scl90 subscales.

	mean	Std. deviation	skewness		kurtosis		Shapiro-wilk		
			statistic	Std.Error	statistic	Std.Error	statistic	df	Sig
De-DASS	13.07	4.80	0.91	0.89	0.92	0.17	0.94	748	<0.001
ANx-DASS	11.35	3.75	0.50	0.89	-0.27	0.17	0.96	748	<0.001
STre-DASS	15.19	4.69	0.89	0.89	0.4	0.17	0.92	748	<0.001
BPQBA	45.41	11.63	0.83	0.89	1.14	0.17	0.96	748	<0.001
BPQSUPR	22.18	7.50	1.59	0.89	2.82	0.17	0.83	748	<0.001
BPQSUBR	11.01	3.72	0.83	0.89	0.76	0.17	0.94	748	<0.001
BPQ	78.60	20.04	0.98	0.89	1.17	0.17	0.94	748	<0.001
DASS	39.62	11.95	0.74	0.89	0.26	0.17	0.95	748	<0.001
SCL90	21.21	7.52	1.06	0.89	0.71	0.17	0.91	748	<0.001

note 1. De-DASS = Depression subscale of DASS. ANx-DASS = anxiety subscale of DASS. STre-DASS = stress subscale of DASS. BPQBA = body awareness subscale of BPQ. BPQSUPR = supra-diaphragmatic reactivity. BPQSUBR = sub-diaphragmatic reactivity. Sig <0.001.

### 3.2Validity

#### Concurrent criterion validity

The DASS and the SCL-90-SOM subscale were used along with the BPQ for concurrent validity purposes. Pearson’s correlation coefficient was calculated among all scales. Since the correlation between two variables can be influenced by the reliability of the tests, the reliability correction for the attenuation formula was used [39]. As seen in Table 7, the BPQ-SF subscales are appropriately and positively correlated with the SOM of SCL-90 and DASS subscales. Each of the two scales used for convergent validity showed a good relationship (0.44–0.94) with each other.

**Table 7:** Concurrent criterion validity whit Pearson Correlation for Body Perception Questionnaire-Short Form (BPQ_SF) Subscales, Depression Anxiety Stress Scale (DASS), Symptom Checklist 90 (SCL_90) and omega and, Cronbach’s Alpha for each subscale.

	DeDASS	ANxDASS	STre-DASS	BPQBA	BPQSUPR	BPQSUBR	BPQ	DASS	SCL90
De-DASS	1	0.766**	0.77**	0.61**	0.65**	0.48**	0.69**	0.9**	0.64**
ANx-DASS	0.76**	1	0.81**	0.48**	0.47**	0.44**	0.54**	0.94**	0.54**
STre-DASS	0.77**	0.81**	1	0.52**	0.53**	0.44**	0.85**	0.93**	0.58**
BPQBA	0.61**	0.48**	0.52**	1	0.66**	0.54**	0.93**	0.57**	0.60**
BPQSUPR	0.65**	0.47**	0.53**	0.66**	1	0.59**	0.86**	0.58**	0.64**
BPQSUBR	0.48**	0.44**	0.44**	0.54**	0.59**	1	0.72**	0.49**	0.54**
BPQ	0.69**	0.51**	0.58**	0.93	0.86**	0.72**	1	0.64**	0.69**
DASS	0.90**	0.94**	0.93**	0.57**	0.58**	0.49**	0.64**	1	0.63**
SCL90	0.64**	0.52**	0.58**	0.6**	0.64**	0.54**	0.69**	0.63**	1
Cronbach’s Alpha	0.87	0.82	0.87	0.90	0.89	0.76	0.94	0.93	0.87
McDonald’s omega				0.90	0.89	0.74	0.93		

### 3.3 Reliability

#### Internal consistency

As shown in Table 7, results of McDonald’s Omega were calculated for the Body Awareness subscale (0.90), Supradiaphragmatic Reactivity (0.89), Subdiaphragmatic Reactivity (0.74), and BPQ-SF total (0/94). Cronbach’s Alpha results were also calculated for the Body Awareness subscale (0.90), Supradiaphragmatic Reactivity (0.89), Subdiaphragmatic Reactivity (0.76), and BPQ-SF total (0.93) (Table 7).

#### Composite Reliability (CR) and AVE

As shown in Table 8, the value of AVE in this study shows that (except for the first one), on average, all factors explain more than 30% of the variance of the items in each subscale. Also, the CR values, which are all higher than 0.8 (except the third case), demonstrate that this scale has moderate and high convergent validity.

**Table 8:** Composite Reliability (CR) and Average Variance Extracted (AVE).

	BA	SUPR	SUBR	BPQ-SF (total)
AVE	0.27	0.45	0.39	0.34
CR	0.89	0.84	0.67	0.95

## 4. Discussion

This study was carried out to determine the validity and reliability of the Persian version of the BPQ-SF (BPQ-P-SF) by calculating its construct validity, internal consistency, and convergent validity.

Principle axis factoring revealed a three-factor structure for this questionnaire which is similar to the results of previous studies on this questionnaire. The Chinese [18] and Italian [17] versions and the findings of Cabrera et al. (2018) on Spanish and American samples also resulted in the three BA, SUPR, and SUBR factors [15]. The factor analysis results indicate that body awareness is well shown by one factor. This factor, which involves the items related to the upper parts or the whole body, can reflect common afferent pathways directed to the skull and spine and ultimately merge in the brain stem, and many of them go to higher brain structures. The autonomic reactivity subscale is divided by sub-diaphragmatic and supra-diaphragmatic factors. Supra-diaphragmatic responses possibly stem from VVC, which is responsible for the initial control of supra-diaphragmatic visceral organs. The strong correlations among BA, SUPR, and SUBR can be due to the feedback loops between the afferent and efferent pathways, and the positive association between SUPR and SUBR can denote the effects of the sympathetic system. In this study, item 41 (I feel like vomiting) was equally placed on the SUPR and SUBR factors, which is consistent with the findings from the Spanish sample in Cabrera et al.’s (2018) study and the findings from the Chinese sample in Wang et al.’s (2020) study. Although there were some changes to the final questionnaire and some items were omitted, this three-factor model was reviewed and approved with the help of a confirmatory factor analysis.

Based on the results of concurrent validity and in line with the findings of Wang et al. (2020), the BPQ-SF is positively and moderately correlated with the SOM subscale [18]. This result can be due to the functioning of the autonomic nervous system. Through the autonomic neural pathways, information from the organs, including organs mentioned in the SOM subscale, actually goes to higher brain structures, and the BPQ-SF has also been developed based on these autonomic pathways. Thus, higher scores on the BPQ point to a higher tendency to experience physical and visceral emotions.

In addition, in line with the findings of Poli et al. (2021), the BPQ-SF scores are positively related to the DASS, indicating that higher scores are associated with higher levels of anxiety, depression, stress, and thus distress. Hence, it shows that symptoms of anxiety, depression, and stress are linked to body perception, which seems in line with neuroscientific findings regarding the role of body perception in depression [4] and anxiety [3].

Our findings present evidence for the validity and reliability of the BPQ-SF in the Iranian population; however, some limitations are suggested to be dealt with in future studies. First, in this study, the validity of the scale has been measured in a non-clinical population, thus future studies can implement this scale in clinical populations to provide further insight regarding its utility. Secondly, the instruments used for validity evaluation in this study were all self-report questionnaires that come with inherent error and bias. Therefore, more objective measures and methodologies such as neuroimaging, or objective tasks such as heart rate accuracy detection can be used to further validate the scope of this scale. Thirdly, further investigations in samples of different demographics can provide more validity for BPQ-SF to be used in different subpopulations.

Although our result may have suffered from our relatively small sample size (20:1), they still show that the Persian version of the BPQ-SF is psychometrically sound to evaluate autonomic reactivity awareness and subjective experiences and provides a valid tool to assess and understand body perception and its role in mental health problems. Body perception is related to awareness, decision-making, self-regulation, and emotional experiences among others. More attention has been given to body perception and its role in the psychopathology of psychological and psychiatric problems, and the validation of BPQ-SF will hopefully contribute to the research and clinical efforts addressing this aspect of our adaptive/maladaptive experience.

## Supporting information

S1 Data Raw questionnaire data of all participants. (XLSX)
